# Navigating cancer treatment and care when living with comorbid dementia: an ethnographic study

**DOI:** 10.1007/s00520-020-05735-z

**Published:** 2020-09-21

**Authors:** Claire Surr, Alys W. Griffiths, Rachael Kelley, Laura Ashley, Fiona Cowdell, Ann Henry, Hayley Inman, Michelle Collinson, Ellen Mason, Amanda Farrin

**Affiliations:** 1 Centre for Dementia Research, School of Health and Community Studies, Leeds Beckett University, Leeds, LS1 3HE UK; 2 School of Social Sciences, Leeds Beckett University, Leeds, UK; 3 Faculty of Health, Education and Life Sciences, Birmingham City University, Birmingham, UK; 4 Clinical Oncology, Leeds Teaching Hospitals NHS Trust, Leeds, UK and School of Medicine, University of Leeds, Leeds, UK; 5 Oncology Services, Bradford Teaching Hospitals NHS Foundation Trust, Bradford, UK; 6 Clinical Trials Research Unit, Institute of Clinical Trials Research, University of Leeds, Leeds, UK

**Keywords:** Alzheimer’s disease, Dementia, Cancer treatment, Ethnography, Environment, Care pathways, Transport

## Abstract

**Objectives:**

The risks of developing cancer and dementia increase as we age; however, this comorbidity remains relatively under-researched. This study reports on the challenges that people affected by comorbid cancer and dementia face when navigating engagement with cancer treatment within secondary care.

**Materials and methods:**

An ethnographic study recruiting 17 people with cancer and dementia, 22 relatives and 19 oncology staff in two UK National Health Service Trusts. Observations (46 h) and informal conversations were conducted during oncology appointments involving people with dementia. Semi-structured interviews (*n* = 37) with people living with cancer and dementia, their relatives and staff working in various roles across oncology services were also carried out. Data were analysed using ethnographically informed thematic analysis.

**Results:**

People with cancer and dementia experienced challenges across three areas of navigating cancer treatment and care: navigating through multiple services, appointments and layers of often complex information; repeatedly navigating transport to and from hospital; and navigating non-dementia-friendly hospital outpatient environments alongside the cognitive problems associated with dementia.

**Conclusions:**

Dementia impacts patients’ abilities to navigate the many practical aspects of attending hospital for cancer treatment and care. This study indicates the importance of addressing ways to improve the experience of travelling to and from the hospital, alongside extending the ongoing efforts to develop ‘dementia-friendly’ hospital in-patient areas and practices, to outpatient departments. Such steps will serve to improve hospital-based cancer treatment and care and more broadly outpatient appointment experiences for people with dementia and their families.

## Introduction

Cancer and dementia have increasing prevalence with age [1, 2] and lead to complex health and care needs and poorer outcomes for those with this comorbidity [3]. Varying estimates have been provided of numbers of people affected by comorbid cancer and dementia (CCD) [4]. Our large UK dataset study recently estimated one in 13 (7.5%) people aged 75+ with a cancer diagnosis also have a dementia diagnosis [5]. Thus, a significant number of patients accessing oncology services have CCD.

The limited research conducted in CCD indicates [3, 4] that people with this dual diagnosis experience reduced likelihood of receiving: cancer screening, staging information, curative treatment and adequate pain management, than those without dementia. They have later diagnosis, lower survival rates [4] and are medically complex, having more comorbid conditions than those with cancer or dementia alone [5]. Few studies have explored direct experiences of cancer treatment in this population. Those conducted found that dementia is poorly identified in oncology services, often limits available treatment options, that oncology staff feel unsure of how to care for this population [6], that dementia brings many complexities to cancer treatment decision-making [7–10] and highlights the important but stressful role family carers play in facilitating successful cancer treatment and management [6, 11, 12]. Further research on the care needs of this population is needed.

This paper explores the challenges of *navigating cancer treatment and care* for people with CCD, their family members and staff working in oncology services. It forms part of a larger study, which aimed to understand the cancer treatment and care experiences of people with CCD [13].

## Materials and methods

### Methods

**Fig. 1:**
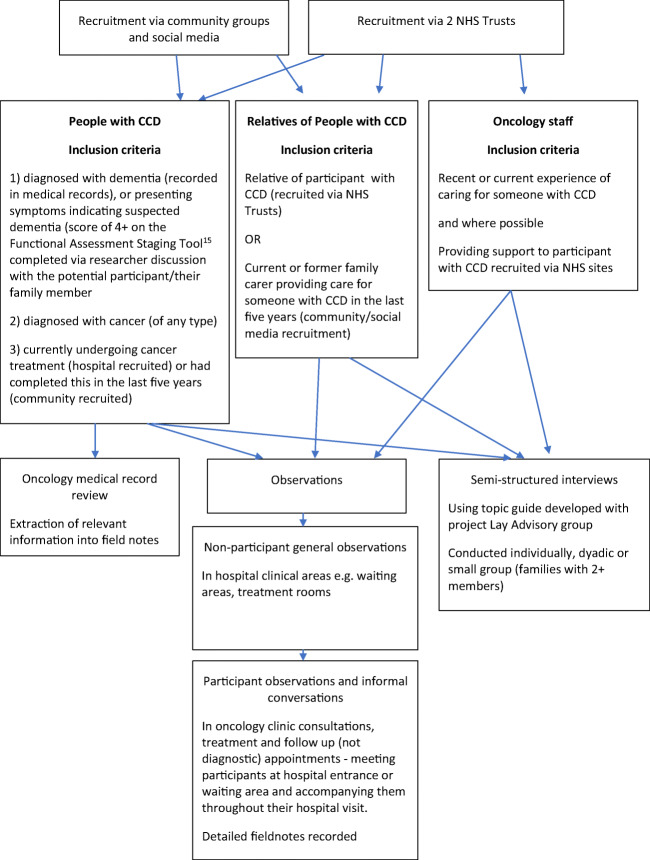
Overview of recruitment and data collection

To gain a rich and nuanced understanding of this relatively unexplored area, we used an ethnographic method including observations, informal conversations and semi-structured interviews (see Fig. 1). All data were collected by RK and AG. General observations (to develop an understanding of routine practices in oncology and radiotherapy departments) were conducted, followed by participant observations where researchers accompanied participants to their oncology appointments. During these, informal conversations were held with participants to explore their ‘in the moment’ perceptions. These data were recorded in detailed written field notes. Observations enabled the inclusion of people with dementia who were unable to participate in formal interviews. Relevant, anonymised information (e.g. references to dementia) was also extracted from participants’ oncology medical records into field notes. Interviews were conducted in private spaces (e.g. participants’ homes, quiet hospital rooms) and explored experiences of cancer treatment and care for people with CCD. They were audio-recorded and transcribed verbatim. People with CCD could participate in observations (if currently receiving cancer treatment at one of the participating hospitals), semi-structured interviews or both, thus maximising the opportunity for people with CCD to participate in the study.

### Participants and sampling

Participants were people with CCD, their families and staff recruited from two English National Health Service (NHS) Trusts, in two cities, providing local cancer services (e.g. surgery, chemotherapy) (both sites) and more specialist regional provision (e.g. radiotherapy) (one site). We also recruited people with CCD who had finished attending hospital regularly for cancer treatment and their families, via local community groups and social media. People could have any type of dementia and degree of severity. We used purposive sampling [14] to recruit participants representing a range of cancer diagnoses, treatment experiences and demographics and staff members working in various oncology roles. Where possible, interviews were conducted with key informants (staff) who had supported participants during observations. Participant inclusion criteria are shown in Fig. 1.

### Lay advisory group

A lay advisory group (LAG) contributed to all aspects of research design and delivery. It included four people affected by cancer and dementia; one person living with CCD and three carers/former carers. One LAG member was also a grant co-applicant.

### Analysis

Data collection and analysis were iterative processes (by CS, AG, RK, FC) using ethnographically informed thematic analysis. Early analysis informed the focus of subsequent data collection. The analysis explored content and patterns in the whole dataset through triangulation across all data sources [15]. Initially, a sample of interview and field note transcripts were read as a whole and then independently coded at a paragraph level, before initial codes were grouped to develop a broad coding framework. A sample of the interview and observational data from people with CCD and family members were initially coded separately to a sample of interviews with staff, with the two coding frameworks subsequently combined. This was continually discussed and refined as further transcripts and field notes were analysed, with input from members of the LAG who also read a selection of transcripts, to produce an overall thematic framework. Definitive themes were finalised through review and discussion, on completion of coding.

### Ethical issues

Ethical approval was gained from the Yorkshire & The Humber – Leeds Bradford Research Ethics Committee ref 18/YH/0145. Written informed consent to participate in interviews and observations was obtained for all participants. Where people with CCD lacked capacity, a personal consultee (relative) was appointed to provide advice on their wishes [16]. Verbal consent was sought from staff present during observations and posters were displayed in observation areas informing patients, staff and other individuals present about the research.

## Results

Seventeen people with CCD, 22 relatives and 19 staff members were recruited, most via the NHS (see Table 1). Data included 37 interviews (person with CCD = 13, relatives = 18, staff = 19; length 9–122 min varying by participant type). Nine hours of non-participant general observations were conducted, alongside participant observations of and informal conversations with 12 people with dementia and the relatives who accompanied them (46.25 h total). Eight people with CCD participated in both observations and an interview.

**Table 1:** Participant demographics

Characteristics	*n* (%)
Participants with CCD (*n* = 17)	
Female, *n* (%)	10 (59)
Cancer type, *n* (%)	
Lung	8 (47)
Prostate	4 (24)
Breast	1 (6)
Gastrointestinal	1 (6)
Other	3 (18)
Ethnicity	
White British	16 (94)
Hispanic	1 (6)
Age, mean (range)* (*n* = 13)	75 (45–88)
Recruitment setting	
NHS	14 (82)
Family caregivers (*n* = 22)	
Female, *n* (%)	14 (64)
Relationship to person with CCD	
Child	12 (55)
Spouse	7 (32)
Sibling	2 (9)
Grandchild	1 (5)
Recruitment setting	
NHS	19 (86)
Staff (*n* = 19)	
Female, *n* (%)	14 (74)
Oncology role worked in	
Radiotherapy dept.	7 (37)
Lung cancer clinic	6 (32)
Breast cancer clinic	3 (16)
Prostate cancer clinic	1 (5)
Other	2 (11)
Staff role	
Nurse	8 (42)
Radiographer	7 (37)
Consultant	2 (11)
Social worker	1 (5)
Patient transport officer	1 (5)

*Two were aged 45–59

The complexity and challenges involved in *navigating cancer treatment and care* alongside having dementia was a major theme identified in the data. Within this, three sub-themes were developed. These were navigating transport; navigating the environment; and navigating services, appointments and information (summarised in Table 2), reflecting navigation challenges across the patient journey.

**Table 2:** Summary of sub-themes

Sub-theme	Theme components
Navigating transport	• Stress of navigating rush hour traffic by car and concerns about arriving on time • Safety concerns around drop-off and parking • Long days and significant waiting times with PTS • Problems with ensuring an escort seat is booked
Navigating the environment	• Difficulties wayfinding to and within out-patient departments • Busyness and noise in waiting areas and departments • Challenges in keeping people with CCD occupied and calm when in waiting areas • Staff recognition of need to improve the oncology environment
Navigating services, appointments and information	• Challenges of managing multiple appointments and remembering required information due to memory problems associated with dementia • Added complexity of additional comorbidities • Need for effective staff communication and connection across multiple services and departments • Heavy reliance of people with CCD on family to support appointment and information navigation

### Navigating transport

Challenges navigating transport to and from oncology appointments were significant and identified in the data of the majority of participants with CCD and several staff interviews. Challenges existed whether people attended the hospital by car or Patient Transport Services (PTS). For those who attended by car, appointment times that required navigation of rush hour traffic extended the length of the hospital visit and added stress, as people worried about arriving on time. Parking necessitated either leaving the person with CCD at busy hospital entrances before parking or having to walk some distance from the car to the department. Both raised safety concerns.

Rush hour traffic when you’ve been there all day… (Site B person with CCD 001)

Thing is, he was… well in to his 80’s he was physically quite well, quite fit. So, we’d find a parking space, sometimes some distance from the hospital and steer towards the hospital. I think sometimes… I don’t know what he’d caught sight of, but he wanted to wander off somewhere else. (Interview Community recruited carer 004 son of man with CCD)

Those using PTS expressed gratitude for the service as they had no other means of transport. However, this entailed long days and significant waiting times, due to early pick-up, or on some occasions anxiety due to transport being late. The PTS vehicle was either a taxi or an ambulance, the latter for those with greater physical needs only. If bookings were not made correctly, carers were not permitted to travel with the person with CCD, causing distress and risks for the person with CCD, PTS and hospital staff. One hospital served a wide geographical region across which local policies differed for the booking of escort seats adding complexity to booking systems.

Patient transport had dropped 001 and 002 off at 1:30PM, despite the appointment not being until 5:25PM ( Observations Site A participant with CCD 001 and her husband 002)

But [man with CCD] came in a taxi one day, transport, without an escort, … they brought him to the wrong place and left him downstairs in reception and we went to find him and there was no sign of him. … The guy on reception said, oh yes, he’s just got a taxi and gone back home (Interview Site A staff participant 008 Lung Clinical Nurse Specialist)

There are certain departments and certain hospitals…[Name] Hospital in [Local suburb] will not allow any escorts whatsoever. So, depending whatever appointment they’re going to, you’re not allowed an escort. Maybe for a dementia patient, but if they were just attending an outpatient appointment … they’re not allowed escorts. (Interview Site A staff participant 0021 Patient Transport Flow Officer)

In one instance, failure to book an escort seat meant the spouse of a participant was not permitted to travel with her to the hospital, causing her significant distress.

Dr [name] asks how she is feeling. 001 replies .. that she has been ‘lost all morning’, saying that she doesn’t know how to describe how she feels ‘don’t know, not worked up’ referring to a ‘problem with the transport place’ that she ‘didn’t know where I was’ and ‘my husband is not with me’. Dr [name] asks whether ‘the transport wouldn’t let him on?’ and she says she has ‘no idea’. It isn’t clear whether 001 has come to the hospital without 002 or whether they arrived at the hospital together and have since become parted. (Observations Site A participant 001 woman with CCD)

Thus, transport to and from the hospital was challenging whichever method was used and could make attendance more stressful for people with CCD and their families. Existing interventions such as provision of drop-off points were not necessarily appropriate for the specific needs of people with CCD.

### Navigating the environment

Having comorbid dementia could make navigating around and waiting within oncology departments difficult. The out-patient departments were often not well-signposted and could be busy and noisy, with many people waiting or walking through. Newly built or refurbished oncology units typically did not appear to have been designed with consideration of dementia-friendly design principles for hospital environments. This was in contrast to in-patient areas of both hospital sites which had implemented dementia-friendly design features.

14:32 Several people were shouting about blood forms, people’s next appointments and ‘I’m doing it now’. The unit was very noisy compared to Friday. (Observations Site B participant 010 woman with CCD)

0010 and I reach the large glass revolving doors and step inside. The glass starts revolving around us – each section of curved glass is separated by a dark divider holding the sections in place, and 0010 steps towards them a couple of times. I realise that she is uncertain which of the glass sections she can actually step out through … she comments on it being difficult to tell where the opening is. (Observations Site A participant 0010 woman with CCD)

Waiting for long periods in oncology departments was exacerbated by comorbid dementia. The impacts of boredom and the weariness that weeks of daily treatment could cause impacted on the emotions and behaviour of the person with CCD. Trying to keep the person occupied and calm in waiting areas not designed for people with dementia could be stressful for carers, some of whom were older and in poor health themselves.

15:55 010’s appointment had now been delayed by almost an hour .. 011: “it’s been an hour, it’s really poor service” 16:12 010 was shuffling about in her seat and puffing her cheeks out “I’ve had enough now, get me out of here.” 16:39 010 “If it’s more than 20 more minutes, I can rebook instead of waiting. I’ve got myself all worked up, I need to leave.” (Observations Site B participant 010 woman CCD and carer participant 011 Sister)

However, staff in some areas had or were in the process of putting in place environmental adaptions to try and address wayfinding and boredom for the benefit of all patients, for example improved signage and use of colours and themed pictures to support conversation, orientation and wayfinding.

So, we’ve got different colours in waiting rooms, and we’re trying to have sort of .. seaside on one, … countryside on next one… So, there’s a bit more, sort of theme, so, if it’s- if you’re seeing pictures of seaside in waiting room, then you’ll see pictures of seaside when you go in for your treatment (Interview Site A staff participant 0042 Radiotherapy Advanced Practitioner)

In summary, the design of hospital out-patient departments was often challenging for people with CCD to navigate and waiting for extended periods was particularly stressful for them and accompanying relatives.

### Navigating services, appointments and information

While receiving cancer treatment, navigating information about diagnosis, treatment and care options and managing multiple appointments can be complex for all patients. Having comorbid dementia and associated memory problems brought additional difficulties in managing these from an organisational and practical perspective.

I would regularly go through things and say, oh you’ve had a letter here from the hospital unopened, and the appointment had come and gone. I had to ring them up and get another appointment (Interview community recruited carer 004 Son of man with CCD)

Hospital appointments for cancer treatment were confusing as they often involved multiple departments and procedures. Remembering medical information to relay to staff and following the instructions associated with treatment was often difficult for people with CCD.

9:13AM 001 and 002 arrived back from the X-rays. 002 told me “It’s bewildering this place. We went down this morning where we usually go and he told us to come up here.” 001 was holding an envelope that a blood sample should be put into. She kept looking at the envelope and seemed confused about what it was. She repeatedly showed it to 002. “Should I give this to the woman on the desk?” “No she gave you it.” [They go into consultation appointment] … “Dr: you went for an X-ray?” “001: “No” “Dr: I think you did” “001: I don’t think that was me”. (Observations Site A participant 001 person with CCD and participant 002 husband)

The additional comorbidities experienced by many people with CCD, and associated healthcare appointments, added further complexity.

It’s actually throwing me [having lots of appointments] because there’s all them. It gets that I don’t know where I am some days with it. I mean, I think I’ve pre-op next week at Hospital 2 … Then I’ve got Hospital 3 for my eyes. They want me to register as partially sighted. (Interview Site B, participant 009 man with CCD who lived alone)

Supporting people with dementia to navigate cancer services required staff to effectively communicate and connect care across multiple services and departments. Sometimes this worked successfully and in other cases disjointed working added to the stress of trying to manage two significant conditions.

… once I mentioned to [oncology Doctor] we’d been [to the memory assessment service (MAS)] and got this problem [dementia diagnosis]. Erm, he actually got in touch with [MAS doctor] to find out what medication … she was going to supply because … he wanted to know exactly what it was. So it wouldn’t interfere with anything he was doing (Interview Site A carer participant 0024 husband of woman with CCD)

One department didn’t talk to the other, so you’d go down to radiotherapy, because she had to have radiotherapy twice a day... and said “well we have to have it quickly because she’s starting on chemo in half an hour”, they didn’t seem to understand why they hadn’t been told about that (Interview Site B carer participant 002 husband of woman with CCD)

People with CCD often relied heavily on family members to support navigation around oncology departments and services. Where this support was not available it was unclear who else could provide this. People with CCD could easily ‘fall between the gap’ without goodwill and flexibility from staff to support their attendance.

.. they [appointment letters] come to dad but then he leaves them out for me and he will say. I’ve got a letter from the hospital and he says oh have a look when you come up. (Interview Site A participant 0040 daughter of man with CCD)

I think we all, to a degree, bend the rules a little bit and work together to do it. … I couldn’t find anywhere to find that support for someone to escort him [man with CCD]. It just didn’t exist. (Interview Site A staff participant 0013 Social worker of man with CCD)

In summary, the memory problems associated with dementia made navigation of appointments and provision of required medical information more complex. There was significant reliance on family members to successfully navigate attendance and on oncology staff to effectively connect multiple services together, work flexibly and support unaccompanied people with CCD.

## Discussion

Our study has offered significant new insights into the challenges people with dementia and their family members and staff supporting them face when navigating attendance at a hospital for cancer treatment and care. There has been extensive progress in improving the care of people with dementia admitted to acute hospitals [17] including consideration of how care is delivered [18–20], staff training on dementia [21, 22] and the suitability of the physical environment [23]. However, to date, this has focussed predominantly on in-patient settings [24] and thus has largely not included out-patient areas including oncology departments.

The impact of multimorbidity on patient and caregiver ability to manage multiple healthcare teams and appointments is well recognised [25]. While multimorbidity is recognised as an important consideration within cancer treatment decision-making [26], particularly in older people, few oncology interventions target this area and in particular, dementia as a comorbidity [27], and often people with dementia are excluded from taking part in research based on their diagnosis [28]. This study has shown that dementia as a comorbidity requires specific considerations over and above those of managing multimorbidity alongside cancer more generally. In particular, oncology services need to recognise that the memory problems associated with dementia lead to a range of difficulties managing appointments, understanding information about diagnosis, treatment and care options, which is further exacerbated by additional comorbidities. Family members are crucial to successful navigation and this study has also identified that those without family to fulfil this role may fall between the gaps [12].

Our study also identified the challenges that the physical environment and extended periods of waiting in oncology departments brought for people affected by dementia. The broader oncology literature recognises the importance of the physical environment and its design to experiences of cancer treatment and care, the impact this may have for patient well-being, safety and recovery [29–31]. It has also begun to recognise that specific groups of patients may have different perceptions and needs associated with the physical environment in which cancer services are delivered [32, 33]. The radiotherapy literature discusses provision of individualised care and adjustments being made to support people with learning disabilities to receive radiotherapy [34, 35]. However, with the exception of ward environments during in-patient stays [36], the oncology literature has not extended to consider the specific needs of people with dementia. The importance and features of dementia-friendly hospital in-patient environments are well-established [37, 38]. This evidence suggests relatively small changes to signage, colour schemes, artwork and provision for activity and engagement can improve patient and carer experience [37]. Additionally, this work recognises the important role that families play in supporting people and of facilitating them to accompany/stay with the person during their time in hospital [39]. Our research indicates the importance of expanding this to include oncology departments and other hospital out-patient departments, as these services support considerable numbers of patients with dementia, many of whom attend regular clinics, often over an extended period of time.

Our study has also indicated that the ‘dementia friendliness’ of oncology services needs to consider people’s experiences of travelling to and from these services. This important aspect of patient and carer experience of hospital attendance has largely been unexplored within both the dementia and oncology literature [40]. The challenges of transportation were experienced whether people attended hospital by car or PTS. Participants identified a range of solutions to make attendance by car easier, including ‘meet and greet’ support at drop-off points and reserved parking spaces for people with dementia close to oncology departments. Identified mechanisms to address difficulties associated with travel via PTS included improved recording of dementia and individual needs on booking forms and greater consistency in escort policies across the geographical areas served by one oncology service. However, wider consideration of how PTS’s might be improved to meet the needs of people with dementia requires further robust exploration, due to the complexity and high user volume of the service, and the little or no research in this area to date [41, 42].

This study is one of a limited number to explore the cancer care and support needs of people with dementia and a range of cancers, their relatives and oncology staff. Limitations include a relatively small sample of largely white, British participants, which, whilst recruited from more than one NHS Trust, all reside in one geographical area of the UK. The sample also lacked the diversity of cancer types and treatments accessed, with a predominance of participants with lung and prostate cancer and out-patient-based treatments such as radiotherapy, with under-representation of those receiving surgery. It is also likely that our study did not include the experiences of people with milder, undiagnosed dementia, who may not have been detected via our screening process using the FAST. Future research may benefit from gaining a deeper understanding of how the stage of cancer and degree of dementia severity impact cancer treatment and care experiences for people with dementia, their families and oncology staff.

In summary, people with dementia and their carers require additional time and support to successfully navigate oncology appointments. Work already ongoing to improve in-patient care to people with dementia in acute hospital settings needs to be extended to oncology and other out-patient departments. These include adapting the physical environment to be more ‘dementia friendly’, more consistent involvement of families in care and consideration of ways to address identified challenges in transport to and from hospital.

## Data Availability

Data may be made available on reasonable request to the corresponding author for the purposes of further research.
